# INPP4B suppresses prostate cancer cell invasion

**DOI:** 10.1186/s12964-014-0061-y

**Published:** 2014-09-25

**Authors:** Myles C Hodgson, Elena I Deryugina, Egla Suarez, Sandra M Lopez, Dong Lin, Hui Xue, Ivan P Gorlov, Yuzhuo Wang, Irina U Agoulnik

**Affiliations:** Florida International University, Miami, Florida USA; The Scripps Research Institute, La Jolla, California USA; Vancouver Prostate Centre & Department of Urologic Sciences, University of British Columbia, Vancouver, BC Canada; Dartmouth College, Hanover, New Hampshire USA; Department of Experimental Therapeutics, BC Cancer Agency, Vancouver, BC Canada; Baylor College of Medicine, Houston, Texas USA

**Keywords:** INPP4B, Invasion, Prostate cancer, Protein kinase C, Interleukin 8, Survivin/BIRC5

## Abstract

**Background:**

INPP4B and PTEN dual specificity phosphatases are frequently lost during progression of prostate cancer to metastatic disease. We and others have previously shown that loss of INPP4B expression correlates with poor prognosis in multiple malignancies and with metastatic spread in prostate cancer.

**Results:**

We demonstrate that *de novo* expression of INPP4B in highly invasive human prostate carcinoma PC-3 cells suppresses their invasion both *in vitro* and *in vivo*. Using global gene expression analysis, we found that INPP4B regulates a number of genes associated with cell adhesion, the extracellular matrix, and the cytoskeleton. Importantly, *de novo* expressed INPP4B suppressed the proinflammatory chemokine IL-8 and induced PAK6. These genes were regulated in a reciprocal manner following downregulation of INPP4B in the independently derived INPP4B-positive LNCaP prostate cancer cell line. Inhibition of PI3K/Akt pathway, which is highly active in both PC-3 and LNCaP cells, did not reproduce INPP4B mediated suppression of IL-8 mRNA expression in either cell type. In contrast, inhibition of PKC signaling phenocopied INPP4B-mediated inhibitory effect on IL-8 in either prostate cancer cell line. In PC-3 cells, INPP4B overexpression caused a decline in the level of metastases associated BIRC5 protein, phosphorylation of PKC, and expression of the common PKC and IL-8 downstream target, COX-2. Reciprocally, COX-2 expression was increased in LNCaP cells following depletion of endogenous INPP4B.

**Conclusion:**

Taken together, we discovered that INPP4B is a novel suppressor of oncogenic PKC signaling, further emphasizing the role of INPP4B in maintaining normal physiology of the prostate epithelium and suppressing metastatic potential of prostate tumors.

**Electronic supplementary material:**

The online version of this article (doi:10.1186/s12964-014-0061-y) contains supplementary material, which is available to authorized users.

## Background

Death from prostate cancer is invariably preceded by metastasis. Approximately 85-100% of terminal cases involve metastases to bone [1] and other organs. At the time of diagnosis, disseminated prostate cancer cells are frequently present at distant sites [2,3]. Understanding the pathways that regulate the metastatic process could facilitate identification of novel therapeutic targets to manage advanced prostate cancer.

While castration therapy remains the primary treatment option for patients with metastatic prostate cancer, such therapies frequently fail and patients recur with castration-resistant metastatic disease. This suggests that castration itself activates pathways that lead to prostate cancer recurrence and metastases [4]. Taylor *et al.* demonstrated that all prostate cancer metastases that develop after androgen ablation have activated PI3K/Akt signaling [5]. In normal prostate epithelium and primary tumors, Akt signaling is suppressed by inositol polyphosphate 4-phosphatase type II (INPP4B) and Phosphatase and Tensin homolog deleted on chromosome 10 (PTEN), which are lost in 47% and 42% of metastases, respectively [5]. In our previous report, we demonstrated that AR directly regulates expression of INPP4B in prostate cancer cells, suggesting that castration may lead to a decline in INPP4B and activation of Akt signaling [6].

Similar to PTEN, INPP4B is a dual specificity phosphatase. INPP4B dephosphorylates phosphatidylinositol polyphosphates on the 4^th^ position of the inositol ring and has phosphotyrosine phosphatase activity [7]. Three known substrates of INPP4B are inositol-1,3,4-trisphosphate (Ins(1,3,4)P3), phosphatydylinositol-3,4-bisphosphate (PI(3,4)P2), and phosphatidylinositol-4,5-bisphosphate (PI(4,5)P2) [8,9]. PI(3,4)P2 binds to the pleckstrin homology domains of Akt and PDK1 and recruits them to the plasma membrane, activating Akt. PI(3,4)P2 is present at low levels on the cell membrane and accumulates at the site of invadopodia [10], specialized structures formed in invasive cells [11-14]. The INPP4B substrate PI(4,5)P2 is the most abundant among the protein-interacting phosphoinositides in the plasma membrane [15]. PI(4,5)P2 binds to several proteins that coordinate actin polymerization, such as villin, gelsolin, cortexillin, and cortactin [16-19]. These proteins regulate assembly of podosomes, invadopodia, and lamellipodia, all of which are involved in cellular interactions with the environment, invasion, and motility. In addition, phospholipase C (PLC) hydrolyses PI(4,5)P2 to I(1,4,5)P3 and diacyl glycerol (DAG), which activates PKC signaling and is implicated in tumor metastases [20,21]. Thus, the substrates of INPP4B lipid phosphatase action, PI(3,4)P2 and PI(4,5)P2, are important second messengers in pathways that stimulate prostate cancer invasion.

In the present study, we provide the first evidence that INPP4B suppresses PKC signaling in both androgen-independent PC-3 cells and androgen-sensitive LNCaP cells. We show that INPP4B expression causes downregulation of PKC signaling, which in turn lowers expression of the proinflammatory cytokine IL-8 and its downstream target COX-2. Therefore, loss of INPP4B during prostate cancer progression may cause stimulation of multiple oncogenic signaling pathways, which facilitate tumor cell invasion and metastatic spread.

## Results

### Cellular localization and activity of *de novo* expressed INPP4B

**Figure 1:**
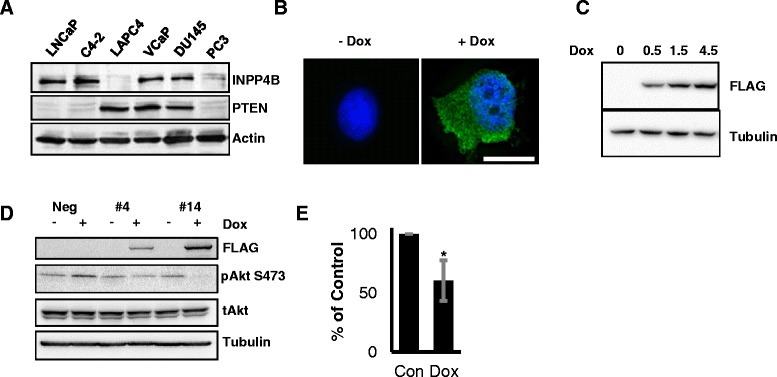
**Induction and localization of INPP4B in PC-3 cells. (A)** Human prostate cancer cell lines were cultured in complete growth media, protein extracted and analyzed for INPP4B, PTEN and actin by Western blotting. **(B)** PC-3 clone #14 cells were cultured for 2 days in the presence or absence of 0.5 μg/ml doxycycline (Dox) in complete medium. Cells were fixed and stained with anti-FLAG M2 antibody, followed by staining with Alexa Fluor 488-conjugated rabbit anti-mouse secondary antibody. Nuclei were counterstained with DAPI. Scale bar = 20 μm **(C)** PC-3 clone #4 with moderate expression of INPP4B was cultured with increasing concentrations of doxycycline and the expression of FLAG-INPP4B and tubulin was analyzed by Western blotting. **(D)** Negative control, #4, and #14 clones were induced with 0.5 μg/ml Dox or left untreated. Protein lysates were analyzed for the levels of FLAG-INPP4B, phospho-Akt (S473), total Akt, and tubulin. **(E)** Using Carestream Molecular Imaging software, band intensities for pAkt 473 and total Akt were determined and p-Akt/Act ratios calculated in four independent experiments for clone #14. Values for pAkt/tAkt in untreated cells were assigned 100% and values for Dox treated cells adjusted proportionally. Percentage of remaining Akt activity in Dox treated cells was averaged for four independent experiments (p = 0.0191).

PC-3 is an invasive human prostate cancer cell line that has the lowest levels of PTEN and INPP4B expression in the tested panel of six prostate cancer cell lines (Figure 1A). We generated several independent PC-3 clones that inducibly express 3xFLAG-INPP4B. In these cells, INPP4B was localized predominantly as speckles on the cellular membrane and to some degree in the cytoplasm (Figure 1B). We observed no INPP4B expression in the absence of doxycycline using either western blotting or immunofluorescence (Figure 1B and C). We chose clones #4 and #14 because they displayed significantly different levels of INPP4B after induction with the same concentration of doxycycline (Figure 1D). Stable cell lines which did not express INPP4B upon induction (Neg) were used as controls (Figure 1D). Since INPP4B can dephosphorylate the membrane phospholipid PI(3,4)P2 [6,9], we tested whether doxycycline induction of INPP4B would inhibit Akt phosphorylation and activation. *De novo* expression of INPP4B significantly reduced serine 473 phosphorylation of Akt (Figure 1D) in PC-3 clone #14 (Figure 1E), but not in clone #4, suggesting that high levels of INPP4B are required to suppress Akt signaling, which is highly active in PC-3 cells.

### INPP4B specifically inhibits prostate cancer cell invasion

**Figure 2:**
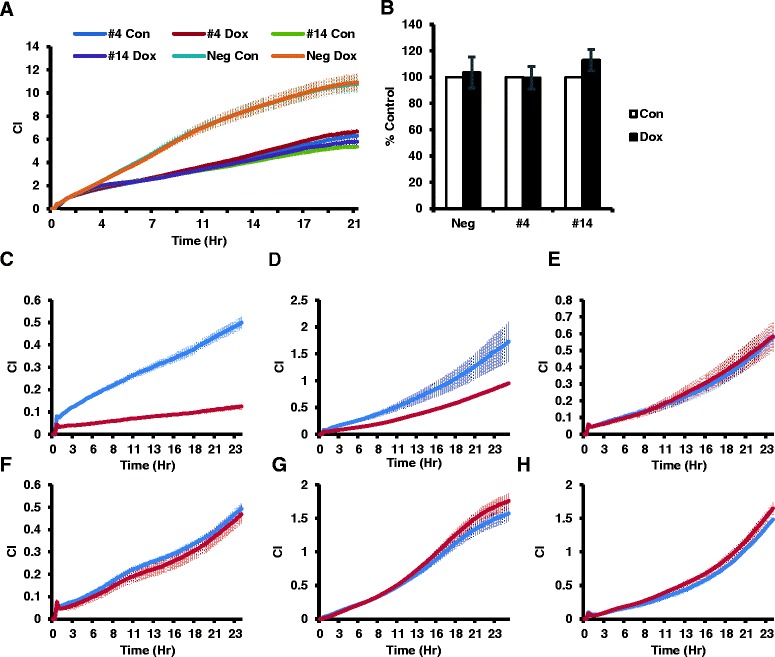
**INPP4B specifically suppresses PC-3 invasion** ***in vitro*** **. (A)** PC-3 Tet-On clones were cultured for 2 days ± 0.5 μg/ml Dox in full growth medium, followed by 24 hours without Dox as described in the methods section. Cells were seeded at 1.5×10^4^ cells per well of an E-Plate 16 and cellular impedance (CI) as a measure of proliferation was monitored continuously for 24 hours. **(B)** Cells were cultured as indicated in **(A)**, and proliferation was compared using MTT assay. The data are presented as percentage of control (means ± SEM) from three independent experiments. Invasion: **(C-E)** Cells were cultured as described in **(A)** and 5×10^4^ cells were seeded onto Matrigel-coated CIM 16 plates and full serum was used as the chemoattractant. Cellular impedance (CI) as a measure of invasion, was monitored continuously for 24 hours for clone #14 **(C)**, clone #4 **(D)** and negative control **(E)**. Migration: **(F-H)** Cells were cultured as described in **(A)** and 5×10^4^ cells seeded onto CIM 16 plates without Matrigel. CI, as a measure of migration was monitored continuously for 24 hours for clone #14 **(F)**, clone #4 **(G)** and negative control **(H)**. Blue lines denote untreated cells and red lines denote doxycycline-treated cells.

We next examined which functions of PC-3 cells were specifically affected after induction of INPP4B. INPP4B expression in PC-3 clones #4 and #14 did not affect their proliferation, as measured by xCELLigence RTCA or MTT assays (Figure 2A and B, respectively). Doxycycline-induced expression of INPP4B significantly reduced PC-3 cell invasion through Matrigel for both clone #14 and clone #4 (Figure 2C and D). In contrast, chemotactic migration of PC-3 cells was not affected by INPP4B expression (Figure 2F and G). Importantly, proliferation, migration, and invasion were not altered by doxycycline treatment of control PC-3 cells that do not express inducible INPP4B (Figure 2A, E, and H). To determine whether hapotactic migration was altered by INPP4B, the outer membranes of xCelligence CIM plates were coated with either fibronectin or collagen prior to cell motility assays. As seen from Additional file 1, INPP4B expression did not change the rates of haptotactic cell migration. These results indicate that INPP4B selectively suppresses invasion of PC-3 cells without significantly affecting their proliferation or migration.

### INPP4B suppresses invasion *in vivo*

**Figure 3:**
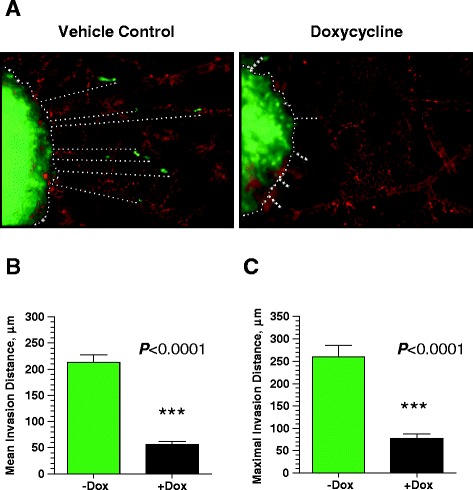
**INPP4B suppresses PC-3 invasion** ***in vivo*** **. (A)** PC-3 cells expressing inducible INPP4B (Clone #14) were incubated in the presence or absence of doxycycline (Dox) and labeled with CellTracker Green. Single cell suspension (~5 μL of 2.5×106 cell/mL) was inoculated into the CAM mesoderm of day 10 embryos developing *ex ovo*. On day 6 after inoculations, microtumor bearing embryos were injected with Rhodamine-conjugated LCA to highlight the CAM vasculature (red). Portions of the CAM containing microtumors were excised and imaged. Microtumor borders and invasion distances of green fluorescent tumor cells are indicated by white dotted lines. Scale bar, 50 μm. **(B)** Quantification of mean invasion distance. **(C)** Quantification of maximal invasion distance (mean of 3 maximal invasion distances). One of two independent experiments is shown. Quantitative analysis was performed on 9-16 microtumors, with 3-5 embryos per variant. Data are presented as means ± SEM. ***P < 0.0001, two-tailed Student’s t test.

To validate our *in vitro* observations that INPP4B suppresses PC-3 cell invasion, we employed a chick embryo model [22]. In this model system, fluorescently-labeled tumor cells were injected directly into the mesoderm layer of the chorioallantoic membrane (CAM) of chick embryos developing *ex ovo*. Five to six days after injection, embryos bearing microtumors were inoculated with contrasting fluorescent lectin to highlight the CAM vasculature. Tumor cell escape and invasion into the mesoderm were then visualized with an epifluorescent microscope and quantified in digitally acquired images. As shown in Figure 3A, control PC-3 cells appear to escape from the primary microtumor and invade the mesodermal stroma. However, induction of INPP4B expression in clone #14 resulted in significant suppression of stromal invasion. Quantification of distances traveled by tumor cells that escaped from primary microtumors demonstrated a 70% inhibition of overall stromal invasion (Figure 3B) and a similar 73% inhibition of maximal invasion distance by individual cells (Figure 3C). These *in vivo* data confirmed our *in vitro* findings that INPP4B suppresses prostate cancer invasion.

### INPP4B expression does not suppress invasion-promoting proteases

Proteases remodeling extracellular matrix (ECM) play a significant role in prostate cancer invasion. Therefore, we investigated whether INPP4B-mediated inhibition of PC-3 cell invasion could be attributed to reduced secretion of matrix metalloproteinases (MMPs) and serine proteases. Conditioned media and cellular extracts from PC-3 cells, with and without INPP4B induction, were analyzed for several secreted and intracellular proteases. As evidenced by zymographic analyses, INPP4B induction did not significantly alter expression levels of secreted gelatinases, MMP-2 and MMP-9 (Additional file 2, A-C), or casein-specific proteases (Additional file 2, D-F). Substrate activity assays indicated that the overall proteolytic activities of intracellular cathepsin B and secreted urokinase-type plasminogen activator (uPA) were not affected by INPP4B expression (Additional file 2, G-H). These findings suggest that INPP4B-mediated suppression of PC-3 cell invasion is achieved without a reduction in the overall proteolytic activity of INPP4B-expressing cells.

### INPP4B-mediated gene regulation in PC-3 Tet-On cells

To identify gene pathways regulated by INPP4B in PC-3 cells that could account for the suppression of invasion, we evaluated transcriptome changes following INPP4B induction in clone #14 cells. Using gene ontology (GO) analysis, we identified 268 genes differentially expressed with INPP4B induction (Additional file 3). Statistical analysis showed that genes associated with cell adhesion and the ECM deposition were enriched among differentially expressed genes (Chi. squared test = 3.9, df =1, P =0.03). A total of 21 genes associated with these processes were identified: *ANXA2, ATP1B1, CDH2, COL6A3, CST3, CXCR7, EFNA1, FZD4, GPR56, GPR56, HES1, ITGB1, ITGB5, LAMB3, MMP13, MMP23A, MMP23B, PI3, PPAP2B, PPAP2B, SIRPA*. We also identified a total of 22 differentially expressed genes associated with the cytoskeleton: *ALDOC, BIRC5, CALD1, FLNA, KRT81, RP2, DYNLT1, PDLIM1, KIF20B, MVP, KIF20A, KATNB1, TUBB3, PLK4, MYH15, GABARAPL1, TMEM214, GOLSYN, MLPH, PHLDB2, TRIM9, TTLL11*. However, enrichment of cytoskeleton-associated genes did not reach statistical significance among differentially expressed genes (Chi. Squared test = 0.9, df =1, P = 0.73).

**Figure 4:**
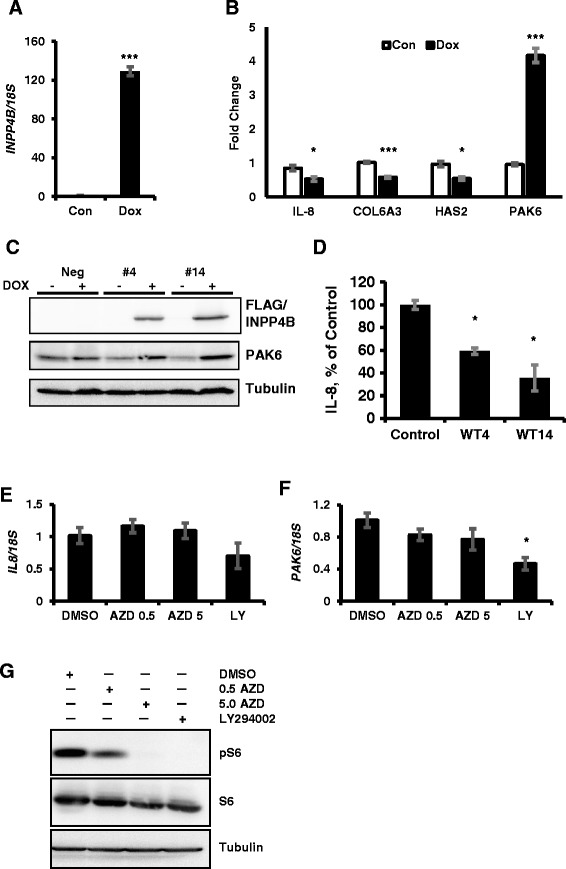
**INPP4B regulation of IL-8 and PAK6 is independent of Akt.** PC-3 Tet-On #14 cells were cultured for 2 days with or without doxycycline on matrigel coated plates in full medium. **(A)** INPP4B induction levels were determined by quantitative RT-PCR. **(B)** Validation of gene expression in samples from **(A)**: IL-8, COL6A3, HAS2 and PAK6. **(C)** Negative control, #4, and #14 PC-3 cell lines were treated as in **(A)** and cellular extracts were analyzed for FLAG-INPP4B, PAK6, and tubulin expression. **(D)** Negative control, #4, and #14 clines were induced with 0.5 μg/ml of doxycycline for 48 hours in complete medium. Conditioned medium was collected, cleared by centrifugation and concentrations of secreted IL-8 protein were determined using human IL-8-specific capture ELISA kit. Values were calculated as a percent of IL8 expression in negative control PC-3 clone. **(E-F)** PC-3 cells cultured in growth medium were treated with DMSO (vehicle), 0.5 μM AZD5363, 5 μM AZD5363 or 10 μM LY294002 to inhibit Akt or PI3K respectively. RNA was extracted and analyzed for expression of *IL-8* **(E)** and *PAK6* **(F)** by quantitative PCR and normalized to *18S*. **(G)** Inhibition of PI3K and Akt by LY294002 and AZD5363 was confirmed by analyzing expression and activation status of the ribosomal protein S6 by Western blot analysis of PC-3 clone #14 cells treated with 0.5 or 5 μM AZD5363 (AZD) and 10 μM LY294002 (LY). Tubulin was used as a loading control. Data are presented as means ± SEM. * *P* < 0.05, **P < 0.01, *** *P* < 0.001.

We validated INPP4B-dependent regulation of gene expression in PC-3 clone #14 for the following genes: interleukin 8 (*IL-8*), collagen type VI alpha 3 (*COL6A3*), hyaluronan synthase 2 (*HAS2*), and p21 protein (Cdc42/Rac) activated kinase 6 (*PAK6*) (Additional file 3). INPP4B-mediated changes in expression of all four genes were confirmed in three independent experiments (Figure 4A and B). In agreement with the changes in mRNA expression, INPP4B increased levels of PAK6 cellular protein and suppressed secretion of IL-8 for both clone #4 and #14 (Figure 4C and D). The effect was stronger in PC-3 clone #14, probably due to higher levels of INPP4B expression compared to clone #4.

### INPP4B regulation of IL-8 and PAK6 is independent of Akt

Lipid substrates of INPP4B are second messengers for multiple signaling pathways, including Akt and PKC [8,9]. Therefore, to determine whether INPP4B inhibits IL-8 and stimulates PAK6 expression by opposing Akt, PKC, both, or neither signaling pathways, we treated PC-3 cells with PI3K/Akt and PKC inhibitors. Inhibition of Akt with AZD5363 and PI3K with LY294002 did not phenocopy the effects of INPP4B on *IL-8* (Figure 4E) and *PAK6* (Figure 4F) expression. Since AZD5363 inhibits Akt without changing its phosphorylation level, we used phosphorylation of the downstream target, S6 ribosomal protein, to confirm that Akt and PI3K activity was suppressed by AZD5363 and LY294002 (Figure 4G). These data indicate that INPP4B regulation of *IL-8* and *PAK6* expression is not mediated by inhibition of PI3K/Akt signaling.

### INPP4B regulates gene expression via inhibition of PKC signaling

**Figure 5:**
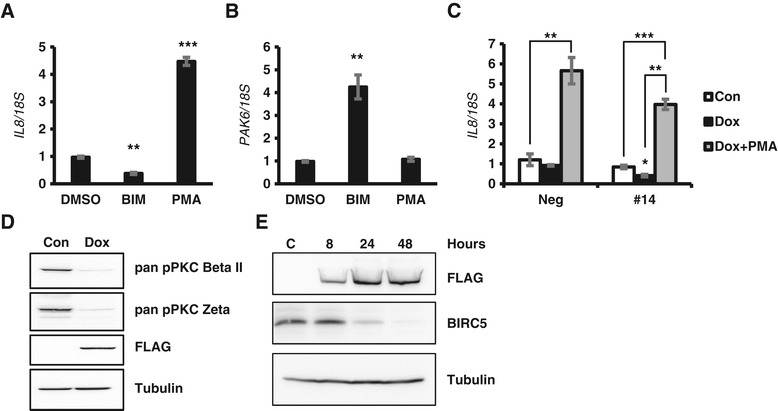
**INPP4B regulates IL-8 and PAK6 through PKC signaling in PC-3 cells. (A-B)** PC-3 cells cultured in regular growth medium were treated with DMSO (vehicle), 2 μM BIM-I (BIM), or 250 nM PMA to inhibit or activate PKC signaling respectively. RNA was extracted and analyzed for expression of *IL-8* **(A)** and *PAK6* **(B)** by quantitative RT-PCR and normalized to *18S*. **(C)** PC-3 Tet-On Neg and #14 clones were cultured for 2 days ± doxycycline and treated with 250 nM PMA for 4.5 hours prior to RNA extraction and gene analysis for *IL-8*. **(D)** PC-3 Tet-On #14 cells were cultured without Dox (Con), or with Dox for 2 days prior to protein extraction. Lysates were analyzed for phospho-PKCβII S660, phospho-PKCζ T410, FLAG-INPP4B, and tubulin. **(E)** PC-3 Tet-On #14 cells were cultured with doxycycline for the indication time periods prior to protein extraction. Lysates were analyzed for BIRC5, FLAG-INPP4B, and tubulin protein levels by Western blot analysis. Data in A, B, and C are presented as means ± SEM.

We next tested whether INPP4B regulates IL-8 and PAK6 by opposing PKC signaling, which is activated by the INPP4B substrate PI(4,5)P2. In parental PC-3 cells, PKC inhibition with bisindolylmaleimide I (BIM-I) decreased expression of *IL-8* (Figure 5A) and increased expression of *PAK6* (Figure 5B). Phorbol 12-myristate 13-acetate (PMA) mediated PKC activation induced *IL-8* but did not suppress *PAK6* expression (Figure 5A and B). PMA treatment of PC-3 Neg and #14 lines showed that PMA could circumvent INPP4B suppression of IL-8 (Figure 5C). To confirm INPP4B-mediated inhibition of PKC activity, we examined PKC phosphorylation levels with two pan phospho PKC antibodies detecting PKC phosphorylated on residues homologous to threonine 410 PKC ζ and serine 660 PKCβ II. Induction of INPP4B in clone #14 for two days significantly reduced PKC phosphorylation (Figure 5D). Since PKC inhibition was shown to reduce levels of survivin (BIRC5) in multiple cell models, including PC-3 [23-25], we examined whether INPP4B induction reduced BIRC5 protein levels. As shown in Figure 5E, INPP4B expression significantly reduced BIRC5 protein 24 and 48 hours post-INPP4B induction. However, during this time period, we did not detect significant changes in BIRC5 mRNA levels (data not shown), suggesting an indirect regulation of BIRC5 by INPP4B. A common PKC and IL-8 downstream target gene is *COX-2*. In PC-3 cells, INPP4B expression significantly (65%) reduced basal transcription of *COX-2* mRNA as well as COX-2 protein (Additional file 4). Thus, INPP4B concordantly regulates multiple proteins in the PKC cascade: IL-8, PAK6, BIRC5, and COX-2.

### INPP4B suppresses IL-8 expression in LNCaP cells

**Figure 6:**
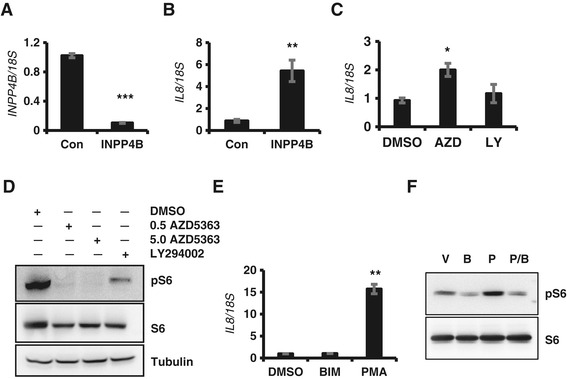
**INPP4B regulates IL-8 gene expression through PKC in LNCaP cells. (A-B)** Regulation of *IL-8* by INPP4B in LNCaP cells was evaluated 48 hours after INPP4B knockdown. RNA was extracted and analyzed for expression of *INPP4B* **(A)** and *IL-8* **(B)** by quantitative RT-PCR and normalized to *18S*. **(C)** LNCaP cells cultured in 10% CSS medium were treated with 5 μM AZD5363 or 10 μM LY294002 for 24 hours. RNA was extracted and analyzed for expression of *IL-8* **(C)** by quantitative RT-PCR and normalized to *18S*. **(D)** Inhibition of PI3K and Akt by LY294002 and AZD5363 was confirmed by western blot analysis of phosphorylation of ribosomal protein S6 following treatment with 0.5 or 5 μM AZD5363 and 10 μM LY294002. **(E)** LNCaP cells cultured in 10% CSS media were treated with BIM-I (BIM) or PMA. RNA was extracted and analyzed for expression of *IL-8* by quantitative RT-PCR. **(F)** LNCaP cells were treated with DMSO **(V)** or 2 μM BIM-I **(B)**, 250 nM PMA **(P)** or BIM-I plus PMA (P/B) to inhibit or activate PKC signaling. Proteins were analyzed for phospho-S6 and total S6 protein levels. Gene expression data are presented as means ± SEM. * *P* < 0.05, **P < 0.01, *** *P* < 0.001.

We have previously shown that INPP4B is endogenously expressed in LNCaP cells where it suppresses Akt signaling [6]. Therefore, we examined whether endogenously expressed INPP4B regulates IL-8 expression and whether this regulation is mediated by Akt or PKC signaling. Consistent with negative regulation of IL-8 by INPP4B, the basal level of IL-8 expression in LNCaP cells is significantly lower than in PC-3 (not shown). Following INPP4B knockdown in LNCaP cells grown in complete medium (Figure 6A), IL-8 expression increased 6-fold (Figure 6B). Growing LNCaP cells in steroid-stripped medium leads to loss of INPP4B and a significant increase in Akt phosphorylation. Under these conditions, PI3K/Akt inhibitors did not reduce IL-8 expression (Figure 6C). Since suppression of Akt and PI3K activity by AZD5363 and LY294002 was confirmed in LNCaP cells by evaluating the phosphorylation status of the S6 ribosomal protein (Figure 6D), these data indicate that in LNCaP cells INPP4B-mediated regulation of IL-8 does not involve PI3K/Akt signaling. Inhibition of PKC signaling had no effect on the low basal level of *IL-8*, while PKC activation with PMA induced *IL-8* expression by 15-fold (Figure 6E). Reciprocal regulation of PKC signaling by BIM-I and PMA was confirmed by examining the phosphorylation status of a PKC downstream target, S6 ribosomal protein (Figure 6F). In agreement with INPP4B inhibition of COX-2 protein in PC-3 cells (Additional figure 4 A-C), depletion of endogenous INPP4B in LNCaP cells resulted in a marked increase in COX-2 levels (Additional figure 4D). Together, these data indicate that in androgen-dependent prostate cancer LNCaP cells, endogenous INPP4B negatively regulates expression of *IL-8* and its target gene *COX-2* at least in part by inhibiting the PKC pathway.

### INPP4B expression is sensitive to castration in patient-derived prostate cancer xenografts

**Figure 7:**
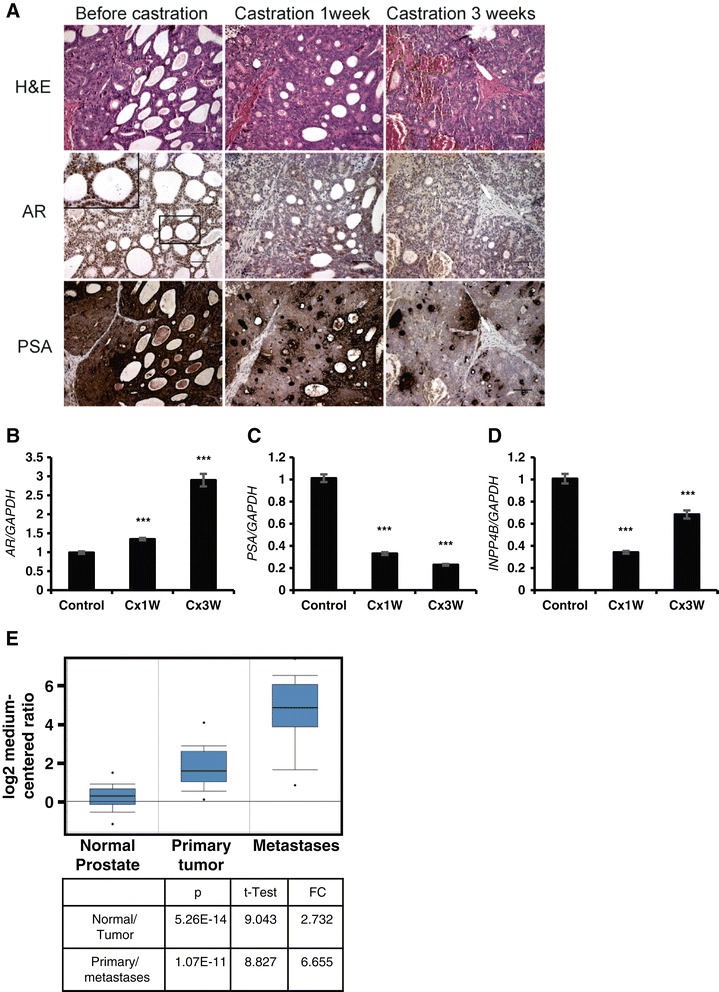
**Decreased PSA and INPP4B expression in a patient tissue derived xenograft line, LTL-418, following castration. (A)** IHC staining shows decreased PSA expression and decreased nuclear expression of AR following castration. Scale bar, 100 μm. **(B-D)** Quantitative PCR shows an increase in *AR* **(B)**, decrease in *PSA* **(C)**, and decrease in *INPP4B* **(D)** expression in LTL-418 xenografts after one (Cx1W) or three (Cx3W) weeks of castration compared to noncastrated control animals. ***P < 0.0001, two-tailed Student’s *t* test. **(E)** Survivn (BIRC5) expression was analyzed using Oncomine and the Grasso Prostate Dataset GSE35988. FC – fold change.

INPP4B is induced by androgens in multiple androgen-sensitive human prostate cancer cell lines [6]. In the absence of clinical samples obtained before and soon after castration, it was not possible to demonstrate androgen regulation of INPP4B in human prostate tumors. To test whether INPPB4 expression is androgen-dependent, we used human prostate cancer xenografts derived from the androgen-responsive, INPP4B-positive xenograft line LTL-418, originally obtained from a high-grade prostate adenocarcinoma [26]. After subrenal capsule implantation, LTL-418 xenografts maintained stromal and glandular epithelial structures characteristic of the original tumor; grew in an acinar pattern, with the majority of the glands fused (Figure 7A). Tumors were grown for 12 weeks prior to surgical castration. In intact animals, LTL-418 tumors are AR and PSA positive (Figure 7A, left panels). Staining for PSA and nuclear AR significantly decreased after castration (Figure 7A, middle and right panels). Gene expression analysis showed that *AR* mRNA steadily increased in response to castration (Figure 7B), while *PSA* mRNA expression and protein levels declined (Figure 7A and C). Expression of *INPP4B* significantly decreased compared to controls at 1 and 3 weeks post-castration, with a slight increase in *INPP4B* expression between week 1 and week 3 after castration (Figure 7D). In LTL-418 tumor xenograft BIRC5 expression was mostly nuclear with a mix of nuclei with high and low staining (Additional file 5A). One and three weeks after castration, practically all nuclei displayed a high level of BIRC5 staining (Additional file 5B and C). Although multiple factors regulate BIRC5 expression, this pattern of *in vivo* expression was consistent with a negative correlation between INPP4B expression and BIRC5 protein in PC-3 cells expressing inducible INPP4B (Figure 5E).

Loss of INPP4B and increased COX-2 and serum IL-8 levels with prostate cancer progression were previously reported [27,28]. Using Oncomine (www.oncomine.org), we examined the Grasso Prostate Cancer Dataset GSE35988 [29] to determine whether *PAK6* and survivin (*BIRC5*) mRNA levels change in prostate cancer compared to normal prostate. In agreement with our cell based studies, *PAK6* expression was lower in prostate cancer (p = 0.003, fold change = -1.350). Remarkably, *BIRC5* expression showed a highly significant increase in both primary (p = 5.26E-14) and especially metastatic (p = 1.07E-11) prostate cancer (Figure 7E). A similar increase in *BIRC5* expression was observed in the Arredouani Prostate Dataset (GSE55945, p = 1.54E-5), the Varambally Prostate Dataset (GSE3325, p = 7.00E-4), the Singh Prostate Dataset (http://www.broadinstitute.org/mpr/publications/projects/Cancer_Susceptibility/references_and_URLS_of_datasets.html, p = 3.36E-4), and other datasets [29-32]. Together, our data strongly suggest that *INPP4B* mRNA expression in human prostate cancer responds to castration, thereby cautioning that androgen ablation therapies might contribute to cancer progression, possibly by reducing INPP4B expression and activation of signaling pathways normally suppressed by INPP4B.

## Discussion

To elucidate metastasis signaling pathways regulated by INPP4B, we used PC-3 cells, an invasive prostate cancer cell line that expresses very low endogenous levels of both PTEN and INPP4B. High de novo expression of INPP4B in PC-3 cells reduced Akt phosphorylation at S473 and activation, albeit to a lesser degree than in LNCaP and VCaP cells [6]. Surprisingly, unlike our previous observation in LNCaP cells [6], INPP4B expression had no impact on the proliferation of two independent PC-3 clones expressing varying levels of INPP4B. This finding is also distinctly different from the functional role of PTEN, which has been shown to suppress proliferation of PC-3 cells [33,34]. Together, these data allows us to suggest that INPP4B and PTEN have unique functions in PC-3 cells and that the anti-proliferative role of INPP4B in LNCaP cells [6] may be linked to AR signaling.

INPP4B is preferentially lost in advanced and metastatic prostate cancer characterized by increased local invasion of tumor cells. [5,6]. We demonstrate that *de novo* expression of INPP4B antagonizes invasion of PC-3 cells *in vitro* without affecting their overall migratory capacity. Confirming our *in vitro* invasion findings, INPP4B severely restricted PC-3 cell invasion into the surrounding stroma in our *in vivo* CAM mesoderm model of stromal invasion. Reciprocally, knockdown of INPP4B increased both invasion and migration of the steroid receptor negative, basal-like MCF10A breast cancer cells [9], suggesting that INPP4B suppression of invasion is common in multiple cancer models.

Cancer cell invasion requires degradation of ECM by numerous tumor and host proteases, including matrix metalloproteinases (MMP) [35]. In particular, MMP-2 and MMP-9 have been implicated in the invasive potential of PC-3 prostate cancer cells [22,36]. Unlike PTEN, that can suppress invasion of PC-3 cells in part through the suppression of MMP activity [37], INPP4B did not inhibit invasion by reducing MMP levels. Other ECM-remodeling proteases, including urokinase plasminogen activator (uPA) and cathepsin B, have been implicated in PC-3 invasion [38-40]. However, INPP4B expression in PC-3 cells reduced neither uPA nor cathepsin B levels. Therefore, our findings apparently exclude impaired matrix proteolysis from the inhibitory effects of INPP4B overexpression on invasion of prostate cancer cells.

To better understand INPP4B-mediated regulation of invasion, we analyzed changes in global gene expression caused by INPP4B overexpression. Among differentially expressed transcripts, genes associated with cell adhesion, ECM, and the cytoskeleton were enriched; all of them associated with invasion. In PC-3 cells, INPP4B also suppressed expression of the proinflammatory chemokine IL-8, both at the mRNA and protein level. IL-8 levels are elevated in the serum of prostate cancer patients with confirmed metastases and in prostate tumor biopsies [28,41,42]. IL-8 has been implicated in tumor progression and induction of reactive stroma in prostate cancer xenograft models [43-46]. IL-8 increases transcriptional activity of the AR and facilitates transition to androgen independence and bicalutamide resistance of prostate cancer [47]. IL-8 is elevated in PTEN deficient prostate cancer cells [48]. Thus, loss of INPP4B in prostate cancers may cause increased IL-8 expression, activation of AR [49], and changes in the tumor microenvironment that lead to prostate cancer progression.

Survivin/BIRC5/API4/EPR-1 is an inhibitor of apoptosis protein (IAP) involved in cell survival, mitosis, stress response and developmental gene expression [50]. Oncomine expression analysis of the Grasso Prostate Dataset reveals that BIRC5 expression is significantly higher in prostate tumors than in the normal prostate gland, with the highest expression in castration resistant metastatic prostate carcinomas [29]. Our new findings demonstrate that INPP4B expression suppresses BIRC5 at the protein level within 24-48 hours of INPP4B expression. Consistent with these observations, BIRC5 was shown to promote invasion of PC-3 cells without inhibiting cell death [51]. BIRC5 stimulates fibronectin expression, β1 integrin signaling, and activation of FAK and Src kinases [51]. It is possible that fibronectin and BIRC5 form a positive feedback loop [51,52]. Fibronectin was not changed in our microarray, but we detected decreased expression of two subunits of the fibronectin receptor, ITGB1 and ITGB5, in INPP4B-expressing cells, suggesting that INPP4B may antagonize β1 integrin signaling.

INPP4B may suppress invasion through suppression of PKC signaling that stimulates IL-8 and BIRC5 expression. We also identified PAK6 as an INPP4B regulated gene in PC-3 cells. The role of PAK6 in prostate cancer is very context dependent, with evidence suggesting that PAK6 can either promote or suppress prostate cancer [53,54]. We show that INPP4B induces PAK6 expression in PC-3, but not in LNCaP cells, possibly due to extremely low basal expression of PAK6 in LNCaP [55].

To determine which signaling pathways are regulated by INPP4B, we utilized Akt, PI3K, and PKC selective inhibitors. Our data shows that inhibition of PKC, rather than Akt or PI3K, replicates key aspects of INPP4B regulated transcription in PC-3 cells. Furthermore, our data suggests that INPP4B inhibits multiple PKC isoforms in PC-3 cells. Significantly, IL-8 signaling has been linked to PLC and PKC activation [56-60] and therefore INPP4B-mediated inhibition of PKC signaling could result in significant downregulation of IL-8. In LNCaP cells, IL-8 expression is barely detectible by quantitative RT-PCR and therefore inhibition of PKC signaling had no effect on IL-8 expression. This suggests that IL-8 expression is at or near basal levels and could not be further inhibited, but was strongly induced by the PKC activator, PMA. Multiple isoforms of PKCs have been implicated in tumor cell proliferation, survival, multidrug resistance, invasion, metastasis and angiogenesis [61]. Specifically, PKC isoforms beta, epsilon, and zeta are elevated during prostate cancer progression and prostate carcinoma invasion [62-64]. One of the downstream targets of PKC/IL-8 signaling is COX-2, a proinflammatory marker associated with prostate cancer invasion [65]. In this study, we have demonstrated that INPP4B overexpression in PC-3 cells inhibited the basal expression of COX-2 and reciprocally, INPP4B knockdown in LNCaP cells elevated COX-2 levels.

Frequent loss of INPP4B in metastases suggests that INPP4B functions to suppress metastatic spread [5,6]. Although AR induction of INPP4B has been demonstrated for androgen-dependent human prostate cancer cell lines [6], it has remained unclear whether INPP4B would decline in clinically-derived human prostate cancer tissue during castration. We evaluated castration sensitivity of INPP4B in the androgen-dependent human prostate cancer LTL-418 xenograft and found that *INPP4B* expression was significantly reduced following castration.

## Conclusions

In conclusion, we demonstrated that INPP4B functions as a suppressor of prostate carcinoma invasion by inhibiting a PKC - IL-8 – Cox-2 – BIRC5 axis, potentially by dephosphorylating or sequestering the lipid substrate PI(4,5)P2, an important second messenger in PLC/PKC signaling. Our data cautions that androgen ablating therapies might promote loss of INPP4B, which in turn could facilitate prostate cancer growth and metastasis through activation of Akt [6] and PKC signaling.

## Methods

### Cell lines and reagents

Human prostate cancer cells, LAPC4, VCaP, DU145, PC-3, and LNCaP cells were obtained from the ATCC (Manassas, VA), which also provided authentication of all cell lines. The cells were grown in ATCC-recommended medium supplemented with 10% fetal bovine serum, 100 U/ml penicillin and 100 μg/ml streptomycin. The authenticated human prostate carcinoma C4-2 was purchased from UroCor. Inc (Oklahoma City, OK) and grown in T medium containing 5% FBS. Cells from each of cell line were frozen at early passages and kept in liquid nitrogen providing a stock for cultured cells that were used within 6 month after thawing. Tetracycline responsive PC-3 cells were maintained in media recommended by ATCC for parental PC-3 cells supplemented with 10% Tet system-approved FBS from Clontech (Mountain View, CA). All culture media were purchased from Life Technologies (Carlsbad, CA); fetal bovine serum (FBS) was purchased from Sigma-Aldrich (St. Louis, MO). Doxycycline (Dox) was from Clontech. Collagen type I, fibronectin, and Matrigel were from Becton Dickinson Biosciences (Bedford, MA). CellTracker™ Green CMFDA was from Life Technologies. Bisindolylmaleimide I (BIM-I) was purchased from AdipoGen (San Diego, CA) and phorbol 12-myristate 13-acetate (PMA) was purchased from Sigma-Aldrich. AZD5363 and LY294002 were purchased from Selleckchem (Houston, TX).

### INPP4B expression constructs

Construction of 3xFLAG N-terminal tagged INPP4B was previously described [6]. To generate a regulated INPP4B expression construct for stable transfection, 3xFLAG-INPP4B was amplified by PCR (forward, 5’- GACAAGCTTGCGGCCGCAGAAATTAAAGAGGAAGGGGC-3’ and reverse 5’- GATGAATTCGCGGCCGCTTAGGTGTCAxGCTTTTCCATAAGTC-3’) and cloned into the *NotI* site of the pLVX-Tight-Puro vector of the Tet-On Advanced expression system (Clontech) to generate pLVX-Tight-Puro- FLAG-INPP4B.

### Construction of INPP4B-inducible cell lines

To produce infectious viral particles, HEK-293 T cells were transiently transfected using the Lentiphos HT/ Lenti-X HT Packaging system with pLVX-Tet-On Advanced lentiviral vector as described by the manufacturer (Clontech). Tet-On PC-3 cells were established by transducing PC-3 cells with Tet-On Advanced lentiviral particles and selecting for stable clones with 500 μg/ml Geneticin. PC-3 Tet-On clones were evaluated for Dox induction by transient transfection with the Dox dependent pLVX-Puro-Luc reporter. The PC-3 Tet-On clone demonstrating the highest level of induction was selected to establish inducible INPP4B clones. PC-3 Tet-On cells were transfected with pLVX-Tight-Puro-FLAG-INPP4B and stable clones selected with 0.5 μg/ml puromycin. Unless otherwise stated, expression of INPP4B was induced by growing cells in the presence of 0.5 μg/ml doxycycline.

### siRNA transfections

LNCaP cells were transfected with siRNA using Lipofectamine RNAiMax (Life Technologies). Briefly, 5×10^5^ cells were transfected with 300 pmol of the indicated siRNA. INPP4B downregulation was performed with silencer siRNAs and noncoding siRNA was used as a control (Life Technologies)[6].

### Western blotting

Cell proteins were extracted with buffer (20 mM Tris-HCl, pH 7.5, 150 mM NaCl, 1 mM EDTA, 1% Triton-X 100), supplemented with protease and phosphatase inhibitors (GeneDepot, Barker, TX). For each sample, 50 μg of protein was resolved on SDS-PAGE and transferred to nitrocellulose membranes. Immunoblotting was performed using the following antibodies: INPP4B 1:1000 (Santa Cruz), FLAG M2 (1:5000) (Sigma-Aldrich), β-Tubulin (1:2000) (Millipore, Billerica, MA), total Akt (1:1000), phospho- S473 Akt (1:1000), COX-2 (1:1000), phospho-S235/236 S6 (1:1000), total S6 (1:1000), survivn/BIRC5 (1:1000), pan phospho-PKC (1:1000) (Cell Signaling Technology, Beverly, MA), PAK6 (1:400) (R&D Systems, Minneapolis, MN). Luminescent signal was captured on a Gel Logic 2000 imaging system with Carestream Molecular Imaging software (Carestream, Rochester, NY).

### Proliferation analysis by MTT assays

PC-3 Tet-On cells cultured for 48 hours with or without 0.5 μg/ml Dox, were seeded at 1×10^3^ cells per well in 96-well culture dishes in medium without doxycycline. After indicated incubation time periods, MTT Reagent (ATCC, MTT Cell Proliferation Assay Kit, Manassas, VA) was added at 10 μl per well and assays performed as suggested by the manufacturer. Absorbance was measured at 570 nm on a FLUOstar Omega (BMG Labtech, Durham, NC) plate reader.

### xCELLigence proliferation, invasion, and motility assays

Proliferation, invasion and migration were performed using Roche DP RTCA xCELLigence analyzer. Analysis of proliferation has been previously described [6]. Migration and invasion were analyzed by first growing cells with or without Dox for 2 days, and then overnight in medium without Dox to eliminate nonspecfic effects of Dox on protease activity. Cell invasion and motility (CIM) plates were coated with 20 μl of Matrigel (Becton Dickinson Biosciences) diluted 1:10 with serum free media for invasion assays, or left uncoated for chemotactic migration experiments. The lower chamber of the CIM plate was filled with 10% Tet-FBS growth media and background impedance determined after equilibrating the plates at 37°C for 1 hour. Cells were plated at 5×10^4^ cells per well and impedance measured every 15 minutes for 24 hours. Impedance is represented by Cell Index (CI) and was calculated as follows: CI = (Z_i_-Z_0_)/15 Ω, where Z_i_ is the impedance at an individual time point and Z_0_ is the background impedance. Average CI was calculated from a minimum of three wells per time point and per experiment.

### xCelligence haptotaxis assay

The undersides of membranes in CIM plates were coated with 10 μg/ml of either fibronectin or collagen I. Plates were coated with collagen I for 3 hours at room temperature, blocked in 5% BSA for one hour, and washed three times with PBS. Fibronectin coating was carried out for 1 hour at 4°C, Followed by 3 washes with PBS, and blocking with 2% BSA. Coated plates were then used in cell motility assay exactly as described above.

### Immunofluorescence

PC-3 Tet-On cells grown on acid etched glass cover slips with or without doxycycline were fixed with 100% ethanol at -20°C, followed by 70% ethanol at -20°C. Background staining was blocked with 5% BSA in PME buffer (400 mM PIPES, pH 6.8, 5 mM EGTA, pH 7.0, and 2 mM MgCl_2_). Cells were incubated with M2 FLAG antibody (1:1000) in 5% BSA in PME buffer overnight at 4°C. Rabbit anti-mouse secondary antibody conjugated to Alexa Fluor 488 were used for M2 FLAG staining and nuclei were counter stained with DAPI (Sigma). Coverslips were mounted on slides with vector shield mounting media (Vector Laboratories). Images were captured on a Zeiss AXIO Imager M2 microscope using Axiovision software (Carl Zeiss MicroImaging GmbH, Munich, Germany).

### Intramesodermal microtumor model for tumor cell escape and stromal invasion

PC-3 cells expressing inducible INPP4B (clone #14) were incubated in the presence or absence of 0.5 μg/ml Dox for 2 days and then labeled with 5 μM CellTracker Green (Molecular Probes, Eugene, OR). Cells were detached, washed, and resuspended at 2×10^6^/ml. Five to seven small boluses of tumor cells (3-5 μl) were injected directly into the CAM mesoderm of day 10 chick embryos incubated *ex ovo* as described [66]. On day 6, tumor-bearing embryos were inoculated with Rhodamine-conjugated *Lens culinaris* agglutinin (LCA; Vector Labs, Burlingame, CA) to highlight the vasculature. Portions of the CAM with microtumors were imaged using a Carl Zeiss AxioImager microscope. Quantification of tumor cell escape and invasion was performed using ImageJ software (NIH, Bethesda, MD). The mean distance of invasion from the microtumor-CAM border was determined for individual microtumors. Maximal invasion distance was determined by averaging 3 maximal distances for individual microtumors. A total of 11 to 13 individual microtumors from 6 to 8 embryos were analyzed for each variable in 2 independent experiments. Data processing and statistical analyses were performed using GraphPad Prism Software (GraphPad Software Inc., San Diego, CA). Statistical significance was evaluated using two-tailed unpaired Student’s *t*-test for *P* < 0.05.

### Zymography

Gelatinase and casein zymography was performed using serum-free conditioned media from PC-3 Tet-On cells that were cultured for 48 hours with and without Dox before overnight incubation in serum-free media. Media were concentrated by centrifugation through Amicon-30 filters (Millipore). Equal amounts of total proteins were used to perform zymography assays as previously described [67]. Zymography gels were photographed on a Gel Logic 2000 imaging system with Carestream Molecular Imaging software (Kodak). Relative density of digested bands was determined using Image J (NIH, Bethesda, MD) software.

### Protease activity assays

Urokinase plaminogen activator (uPA) activity was evaluated in concentrated conditioned media prepared as described above for zymography. uPA activity assays were performed in 100 mM Tris-HCl, pH 7.5, at 37°C with 2.5 mM N-CBZ-Glycyl-Glycyl-L-arginine 7-amido-4-methylcoumarin hydrochloride (N-CBZ-G-G-AMC) for 4 hours. Cathepsin B activity was assayed in cytosolic lysates prepared in 20 mM Tris-HCl, pH 6.8, 1 mM EDTA and 0.1% NP-40. The lysates were incubated in 200 mM NaOAc, 1 mM DTT, with 100 μM Z-Arg-Arg-7-amido-4-methylcoumarin hydrochloride (Z-R-R-AMC) for 4 hours at 37°C. Absorbance was measured at 460 nm on a FLUOstar Omega (BMG Labtech) plate reader.

### RNA preparation and Illumina microarray hybridization

PC-3 Tet-On cells (5×10^5^) were grown in triplicate on Matrigel-coated tissue culture dishes for two days with or without 0.5 μg/ml doxycycline prior to harvesting for RNA extraction. Cells were washed and scraped with ice cold PBS. Total RNA was extracted using Trizol reagent according to the manufacturer’s instructions and two rounds of alcohol precipitation were performed. RNA concentration was determined using a NanoDrop spectrophotometer (Thermo Fisher Scientific, Waltham, MA). The quality of total RNA was assessed by an Agilent 2100 Bioanalyzer using the Eukaryote Total RNA Nano Kit (Agilent Technologies). A total of 500 ng RNA was reverse-transcribed into cRNA and labelled with biotin–uridine triphosphate using the Illumina TotalPrep RNA Amplification Kit. Gene expression was analyzed using the Illumina HumanHT-12v4 expression Beadchip platform. All analyses were performed using GenomeStudio software (Illumina, Inc., San Diego, CA). Background was subtracted and arrays were normalized using quantile. Average signal intensities of samples within the group were used for differential expression analysis.

We also assessed the enrichment of differentially expressed genes for the following functional categories: cell adhesion, extracellular matrix genes, and genes associated with the cytoskeleton. Those functional categories were defined according to the Gene Ontology database [68]. Statistical significance of enrichment was estimated using the Chi. Squared test. Analysis was performed with STATA software (SAS Institute Inc).

### Measurment of IL-8

PC-3 cells were seeded at 5×10^5^ cells per 10 cm plate in complete medium. Cells were induced with 1 μg/ml Dox at for 48 hours. Culture medium was then replaced with 5 mL of culture medium with or without FBS. Conditioned medium was collected 48 hours later and cleared of cell debris by centrifugation at 13,000 g for 5 min at 4°C. Concentrations of secreted IL-8 protein were determined with the human IL-8-specific capture ELISA kit (PeproTech, Rocky Hill, NJ), according to the manufacturer’s instructions. IL-8 production by INPP4B-expressing cell lines was compared to total IL-8 produced by negative control (100%).

### Subrenal capsule grafting

Animal studies were conducted in accordance with the humane standards of animal care, and all procedures were approved by the University of British Columbia Institutional Animal Care and Use Committee. Six- to eight-week old NOD-SCID mice were bred by the BC Cancer Research Centre Animal Resource Centre (BC Cancer Agency, Vancouver, Canada) and used for prostate cancer xenografting. The transplantable xenograft model LTL-418 was developed from a prostatectomy sample of primary prostate adenocarcinoma obtained in accordance with and approved by the Clinical Research Ethics Board of the University of British Columbia (UBC) and the BC Cancer Agency (UBC Ethics board #: H09-01628 and H04-60131; VCHRI #: V09-0320 and V07-0058). The original histopathological and molecular characteristics of the LTL418 model and its response to castration are described elsewhere [26]. Briefly, primary tumor was cut into small pieces (1 × 3 × 3 mm^3^ in size) and grafted under the renal capsule of male NOD-SCID mice supplemented with testosterone as previously described [69]. Testosterone pellets were removed at the time of surgical castration. Tumor tissues were collected prior to castration and 1 week and 3 weeks after castration.

### Histopathology and immunohistochemistry

Preparation of paraffin-embedded tissue sections and immunohistochemical analyses were done as previously described [70]. Rabbit polyclonal antibodies against AR (Affinity BioReagents, Golden, CO), PSA (Dako, Carpinteria, CA), and rabbit monoclonal anti-survivin antibody (#2808, Cell Signaling Technologies, Beverly, MA) were used. Biotinylated anti-rabbit IgGs and peroxidase-linked avidin/biotin complex reagents were obtained from Vector Laboratories (Burlingame, CA). Control sections were processed in parallel with rabbit nonimmune IgG (Dako, Carpinteria, CA) used at the same concentrations as the primary antibodies.

### Quantitative real-time PCR

Total RNA was isolated from xenograft tissues using the RNeasy mini kit (Qiagen, Valencia, CA). The quality of RNA samples was analyzed using an Agilent Bioanalyzer (Agilent Technologies). The cDNA was synthesized using the QuantiTect Reverse Transcription Kit (Qiagen). Expression of selected genes was analyzed using a ViiA™ 7 Real-Time PCR System (Applied Biosystems, San Francisco, CA). The quantitative RT-PCR (qRT-PCR) reaction was carried out in KAPA SYBR® FAST Universal 2X qPCR Master Mix (Kapabiosystems, Woburn, MA). Duplicate reactions were performed for each sample, and data were normalized to GAPDH and averaged. RNA was prepared from cell lines using Trizol reagent (Life Technologies). cDNA was synthesized using the Verso cDNA synthesis kit (Thermo Fisher Scientific). The Roche Universal Probe library was used to amplify selected genes. Real-time qPCR amplification was performed on a Roche 480 LightCycler. See Additional file 6 for primer sequences.

## Additional files

Additional file 1:
**INPP4B expression does not suppress the rate of haptotactic migration.** Outer sides of membranes in the CIM plates were coated with fibronectin (A, C) or collagen I (B, D), blocked with BSA, and rinsed in PBS. PC-3 #14 and #4 cells were treated with either vehicle or 0.5 μg/ml doxycycline for 48 hours. Cells were washed, trypsinized, and plated into CIM plates at 50,000 per well in serum free medium. Full growth medium was used in the lower chamber. Cellular impedance as a measure of haptotactic migration was monitored for 20-30 hours. Additional file 2:
**INPP4B expression does not suppress basal levels of secreted and cellular proteases.** (A-C) PC-3 Tet-On clone #14 and negative for INPP4B clone were cultured for 2 days ± 0.5 μg/ml doxycycline in serum-containing media, followed by incubation for 24 hours without doxycycline in serum-free media as described in Materials and Methods. Conditioned media were collected, concentrated by centrifugation, and analyzed by gelatin zymography (A). Enzyme levels were determined by gelatin digestion in 0.1% gelatin 10% PAGE. Levels of MMP-9 (B) and MMP-2 (C) expression were quantified by densitometry of corresponding bands and normalized to no-doxycycline control for each clone. Bars are means ± SEM determined from 3 independent experiments. (D) Cells were cultured as described in (A) and casein zymography was performed for analysis of casein-cleaving proteases. Expression levels of the ~150-kDa band (E) and ~90-kDa band (F) were quantified by densitometry analysis of the corresponding bands and normalized to no-doxycycline control for each clone. (G) Cathepsin B activity was assayed as described in Materials and Methods, from 10 μg of cell lysate prepared from cells cultured as described in (A). Data from 6 independent experiments were averaged and normalized to untreated cells for each clone (100%). (H) uPA activity was assayed from concentrated media harvested from cells cultured as described in (A) and Materials and Methods. Data from 4 independent experiments were averaged and normalized to untreated cells for each clone (100%). For all graphs, open bars denote untreated cells and closed bars denote doxycycline treated cells. Additional file 3:
**Differentially expressed genes in PC-3 cells following INPP4B induction.**
Additional file 4:
**INPP4B expression inhibits expression of COX-2.** (A and B) PC-3 cells from Tet-On clone #14 and the negative for INPP4B clone were cultured for 2 days ± 0.5 μg/ml doxycycline in full medium. RNA was extracted and analyzed for expression of INPP4B (A) and COX-2 (B) by quantitative PCR and normalized to 18S. Data are presented as means ± SEM. * P < 0.05, ***P < 0.0001, two-tailed Student’s t test. (C) PC-3 control and inducible clones were cultured for 2 days ± 0.5 μg/ml doxycycline in serum-containing media. Proteins were extracted and the expression of FLAG-INPP4B, COX-2, and tubulin was analyzed by Western blotting. Bar graph, Expression of COX-2 (fold-change) was quantified by densitometry relative to tubulin and normalized to no doxycycline for each clone (expressed as 1.0). (D) LNCaP cells were transfected with either noncoding control (Ctrl) or 2 independent INPP4B-specific siRNAs (INPP-1 or INPP-2). Cells were grown for 48 hours in complete medium and cellular protein extracts were analyzed by Western blotting for INPP4B, COX-2 and tubulin. Bar graph, COX-2 protein levels were quantified by densitometry, normalized to tubulin, and fold change in expression levels was determined relative to control siRNA transfected cells (1.0). Data in panels C and D were obtained three times and representative experiment shown. Additional file 5:
**BIRC5 expression increases after castration in LTL-418 xenograft line.** A. BIRC5 IHC staining of LTL-418 xenograft tissue harvested before castration. The staining is nuclear ranging form medium to high intensity. B. BIRC5 staining of LTL-418 xenograft tissue harvested 1 week after castration. C. BIRC levels in LTL-418 xenograft tissue 3 weeks after castration showing high level of staining in absolute majority of nuclei. Additional file 6:
**Quantitative PCR primer sets for gene expression analysis.**

## Abbreviations

Akt: V-akt murine thymoma viral oncogene homolog 1
AR: Androgen receptor
BIM-I: Bisindolylmaleimide I
CAM: Chorioallantoic membrane
CI: Cell index
CIM plates: Cell invasion and motility plates
Con: Control uninduced cells
COX-2: Cyclooxygenase 2
CSS: Charcoal stripped serum
DAG: Diacyl glycerol
Dox: Doxycycline
ECM: Extracellular matrix
FBS: Fetal bovine serum
IL-8: Interleukin 8
INPP4B: Inositol polyphosphate 4-phosphatase type II
Ins(1,3,4)P3: Inositol-1,3,4-trisphosphate
MMP: Matrix metalloproteinase
MTT: 3-(4,5-dimethylthiazol-2-yl)-2,5-diphenyltetrazolium bromide
N-CBZ-G-G-AMC: N-CBZ-Glycyl-Glycyl-L-arginine 7-amido-4-methylcoumarin hydrochloride
Neg: PC-3 Tet-On negative clone (does not express INPP4B)
PAK6: P21 protein (Cdc42/Rac) activated kinase 6
PI(3,4)P2: Phosphatydylinositol-3,4-bisphosphate
PI(4,5)P2: Phosphatidylinositol-4,5-bisphosphate
PI3K: Phosphatidylinositide 3-kinase
PKC: Protein kinase C
PLC: Phopsholipase C
PMA: Phorbol 12-myristate 13-acetate
PSA: Prostate specific antigen
PTEN: Phosphatase and Tensin homolog deleted on chromosome 10
RTCA: Real-time cellular analysis
siRNA: Small interfering ribonucleic acid
Tet: Tetracycline
Z-R-R-AMC: Z-Arg-Arg-7-amido-4-methylcoumarin hydrochloride
