# Supplementary material for: INPP4B suppresses prostate cancer cell invasion
Source: Cell Commun Signal. 2014 Sep 25;12:61. doi: 10.1186/s12964-014-0061-y (PMC4181726; doi:10.1186/s12964-014-0061-y)
Supplement: Additional file 1: — INPP4B expression does not suppress the rate of haptotactic migration. Outer sides of membranes in the CIM plates were coated with fibronectin (A, C) or collagen I (B, D), blocked with BSA, and rinsed in PBS. PC-3 #14 and #4 cells were treated with either vehicle or 0.5 μg/ml doxycycline for 48 hours. Cells were washed, trypsinized, and plated into CIM plates at 50,000 per well in serum free medium. Full growth medium was used in the lower chamber. Cellular impedance as a measure of haptotactic migration was monitored for 20-30 hours. [file 12964_2014_61_MOESM1_ESM.pptx]

## Slide 1
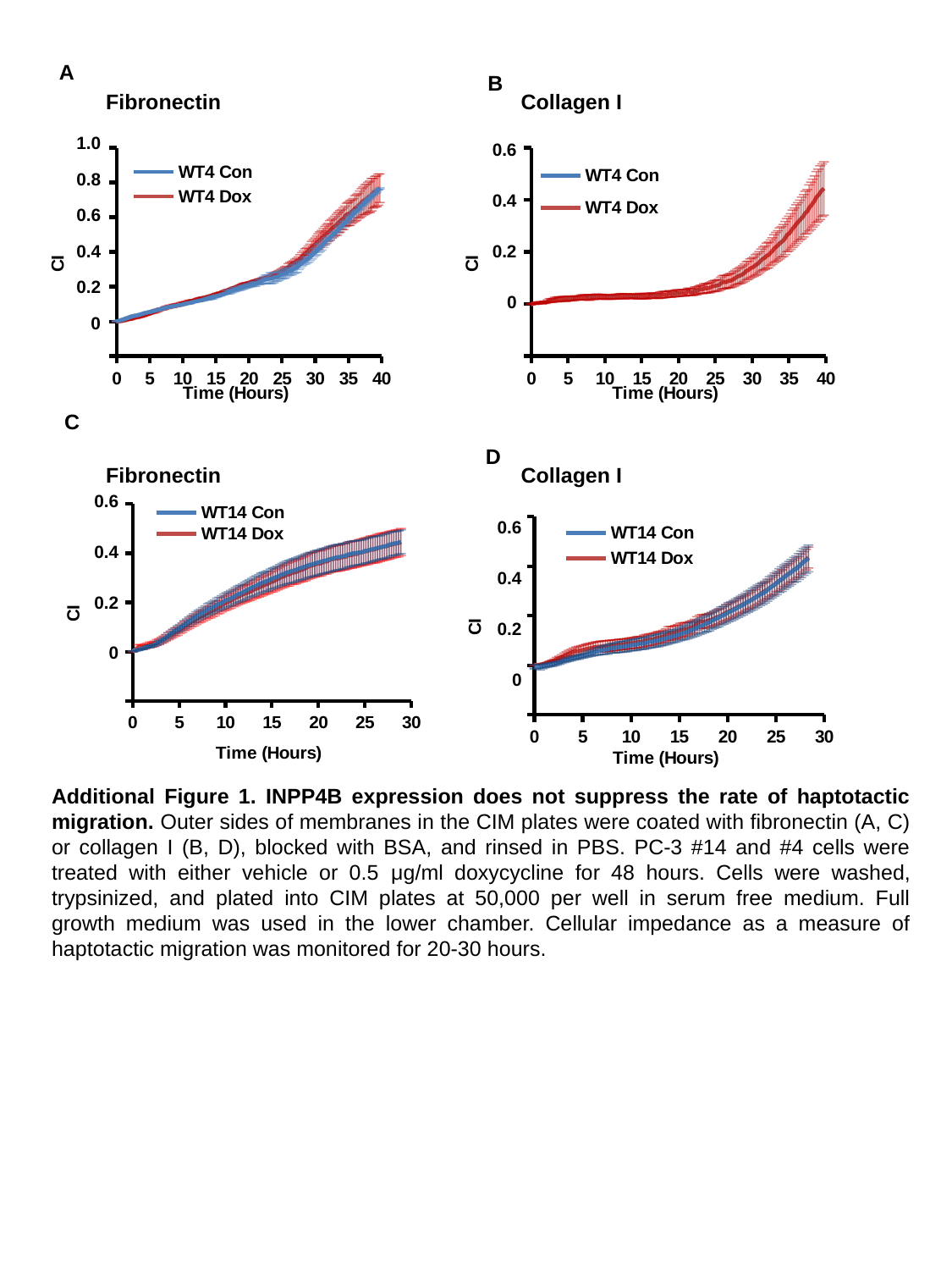

A
B
Fibronectin
Collagen I
1.0
0.8
0.6
0.4
0.2
0
0.6
0.4
0.2
0
### Chart
| Category | WT4 Con | WT4 Dox |
|---|---|---|
### Chart
| Category | WT4 Con | WT4 Dox |
|---|---|---|C
D
Fibronectin
Collagen I
0.6
0.4
0.2
0
### Chart
| Category | WT14 Con | WT14 Dox |
|---|---|---|
### Chart
| Category | WT14 Con | WT14 Dox |
|---|---|---|0.6
0.4
0.2
0
Additional Figure 1. INPP4B expression does not suppress the rate of haptotactic migration. Outer sides of membranes in the CIM plates were coated with fibronectin (A, C) or collagen I (B, D), blocked with BSA, and rinsed in PBS. PC-3 #14 and #4 cells were treated with either vehicle or 0.5 μg/ml doxycycline for 48 hours. Cells were washed, trypsinized, and plated into CIM plates at 50,000 per well in serum free medium. Full growth medium was used in the lower chamber. Cellular impedance as a measure of haptotactic migration was monitored for 20-30 hours.
