# Supplementary material for: INPP4B suppresses prostate cancer cell invasion
Source: Cell Commun Signal. 2014 Sep 25;12:61. doi: 10.1186/s12964-014-0061-y (PMC4181726; doi:10.1186/s12964-014-0061-y)
Supplement: Additional file 2: — INPP4B expression does not suppress basal levels of secreted and cellular proteases. (A-C) PC-3 Tet-On clone #14 and negative for INPP4B clone were cultured for 2 days ± 0.5 μg/ml doxycycline in serum-containing media, followed by incubation for 24 hours without doxycycline in serum-free media as described in Materials and Methods. Conditioned media were collected, concentrated by centrifugation, and analyzed by gelatin zymography (A). Enzyme levels were determined by gelatin digestion in 0.1% gelatin 10% PAGE. Levels of MMP-9 (B) and MMP-2 (C) expression were quantified by densitometry of corresponding bands and normalized to no-doxycycline control for each clone. Bars are means ± SEM determined from 3 independent experiments. (D) Cells were cultured as described in (A) and casein zymography was performed for analysis of casein-cleaving proteases. Expression levels of the ~150-kDa band (E) and ~90-kDa band (F) were quantified by densitometry analysis of the corresponding bands and normalized to no-doxycycline control for each clone. (G) Cathepsin B activity was assayed as described in Materials and Methods, from 10 μg of cell lysate prepared from cells cultured as described in (A). Data from 6 independent experiments were averaged and normalized to untreated cells for each clone (100%). (H) uPA activity was assayed from concentrated media harvested from cells cultured as described in (A) and Materials and Methods. Data from 4 independent experiments were averaged and normalized to untreated cells for each clone (100%). For all graphs, open bars denote untreated cells and closed bars denote doxycycline treated cells. [file 12964_2014_61_MOESM2_ESM.pptx]

## Slide 1
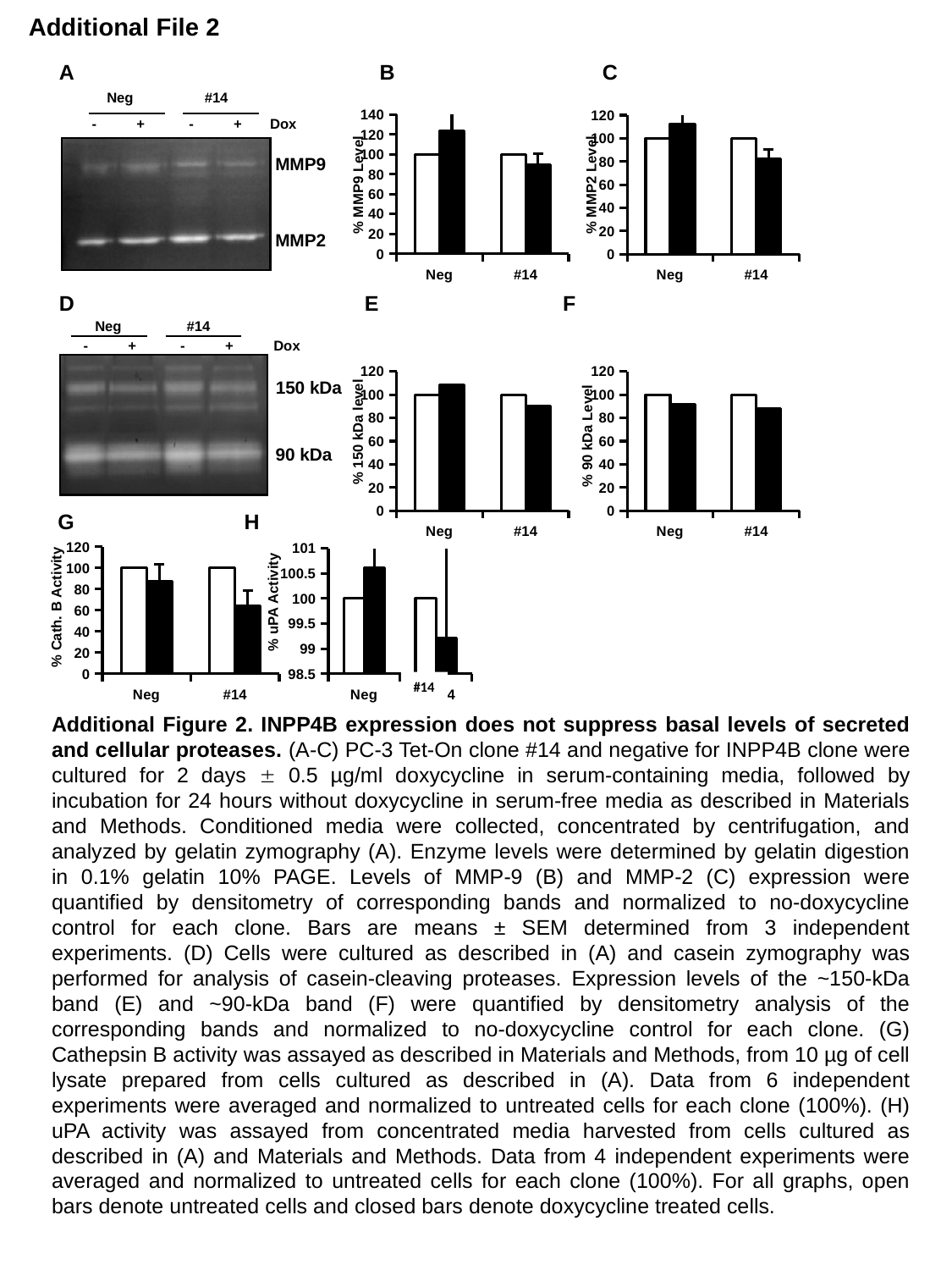

Additional File 2
A
B
C
Neg
#14
### Chart
| Category | Con | Dox |
|---|---|---|
| Neg | 100.0 | 123.74706179860186 |
| #14 | 100.0 | 89.82857083944856 |
### Chart
| Category | Con | Dox |
|---|---|---|
| Neg | 100.0 | 112.3782322138652 |
| #14 | 100.0 | 82.29884417098329 | - + - + Dox
MMP9
% MMP9 Level
% MMP2 Level
MMP2
D
E
F
Neg
#14
 - + - + Dox
### Chart
| Category | Con | Dox |
|---|---|---|
| Neg | 100.0 | 108.41513583981406 |
| #14 | 100.0 | 90.02521307594384 |
### Chart
| Category | Con | Dox |
|---|---|---|
| Neg | 100.0 | 91.43815864767647 |
| #14 | 100.0 | 88.29485782309192 |150 kDa
% 150 kDa level
% 90 kDa Level
90 kDa
G
H
### Chart
| Category | Con | Dox |
|---|---|---|
| Neg | 100.0 | 87.4167035055509 |
| #14 | 100.0 | 64.40845299298435 |
### Chart
| Category | Con | Dox |
|---|---|---|
| Neg | 100.0 | 100.61432655822 |
| WT14 | 100.0 | 99.20736821804425 |% uPA Activity
% Cath. B Activity
#14
Additional Figure 2. INPP4B expression does not suppress basal levels of secreted and cellular proteases. (A-C) PC-3 Tet-On clone #14 and negative for INPP4B clone were cultured for 2 days ± 0.5 µg/ml doxycycline in serum-containing media, followed by incubation for 24 hours without doxycycline in serum-free media as described in Materials and Methods. Conditioned media were collected, concentrated by centrifugation, and analyzed by gelatin zymography (A). Enzyme levels were determined by gelatin digestion in 0.1% gelatin 10% PAGE. Levels of MMP-9 (B) and MMP-2 (C) expression were quantified by densitometry of corresponding bands and normalized to no-doxycycline control for each clone. Bars are means ± SEM determined from 3 independent experiments. (D) Cells were cultured as described in (A) and casein zymography was performed for analysis of casein-cleaving proteases. Expression levels of the ~150-kDa band (E) and ~90-kDa band (F) were quantified by densitometry analysis of the corresponding bands and normalized to no-doxycycline control for each clone. (G) Cathepsin B activity was assayed as described in Materials and Methods, from 10 µg of cell lysate prepared from cells cultured as described in (A). Data from 6 independent experiments were averaged and normalized to untreated cells for each clone (100%). (H) uPA activity was assayed from concentrated media harvested from cells cultured as described in (A) and Materials and Methods. Data from 4 independent experiments were averaged and normalized to untreated cells for each clone (100%). For all graphs, open bars denote untreated cells and closed bars denote doxycycline treated cells.
