# Supplementary material for: INPP4B suppresses prostate cancer cell invasion
Source: Cell Commun Signal. 2014 Sep 25;12:61. doi: 10.1186/s12964-014-0061-y (PMC4181726; doi:10.1186/s12964-014-0061-y)
Supplement: Additional file 4: — INPP4B expression inhibits expression of COX-2. (A and B) PC-3 cells from Tet-On clone #14 and the negative for INPP4B clone were cultured for 2 days ± 0.5 μg/ml doxycycline in full medium. RNA was extracted and analyzed for expression of INPP4B (A) and COX-2 (B) by quantitative PCR and normalized to 18S. Data are presented as means ± SEM. * P < 0.05, ***P < 0.0001, two-tailed Student’s t test. (C) PC-3 control and inducible clones were cultured for 2 days ± 0.5 μg/ml doxycycline in serum-containing media. Proteins were extracted and the expression of FLAG-INPP4B, COX-2, and tubulin was analyzed by Western blotting. Bar graph, Expression of COX-2 (fold-change) was quantified by densitometry relative to tubulin and normalized to no doxycycline for each clone (expressed as 1.0). (D) LNCaP cells were transfected with either noncoding control (Ctrl) or 2 independent INPP4B-specific siRNAs (INPP-1 or INPP-2). Cells were grown for 48 hours in complete medium and cellular protein extracts were analyzed by Western blotting for INPP4B, COX-2 and tubulin. Bar graph, COX-2 protein levels were quantified by densitometry, normalized to tubulin, and fold change in expression levels was determined relative to control siRNA transfected cells (1.0). Data in panels C and D were obtained three times and representative experiment shown. [file 12964_2014_61_MOESM4_ESM.pptx]

## Slide 1
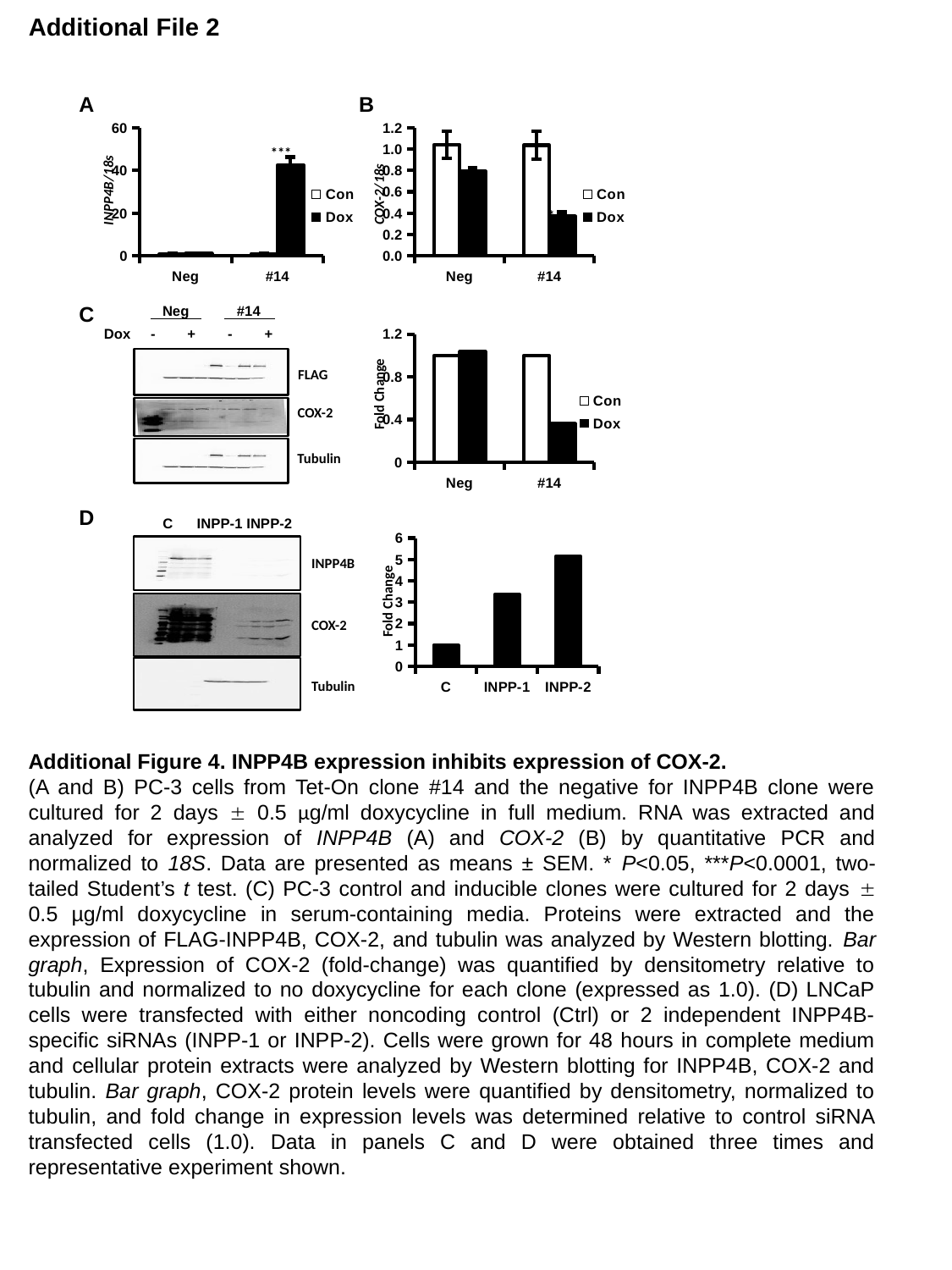

Additional File 2
A
B
### Chart
| Category | Con | Dox |
|---|---|---|
| Neg | 1.043390403308478 | 1.115389743183315 |
| #14 | 1.040171803547848 | 42.48812957469642 |
### Chart
| Category | Con | Dox |
|---|---|---|
| Neg | 1.041440770938872 | 0.79319367414955 |
| #14 | 1.037581814459115 | 0.371970424527677 |***
INPP4B/18s
COX-2/18s
*
C
Neg
#14
Dox - + - +
### Chart
| Category | Con | Dox |
|---|---|---|
| Neg | 1.0 | 1.034842235771403 |
| #14 | 1.0 | 0.362267768739855 |
FLAG
Fold Change
COX-2
Tubulin
D
 C INPP-1 INPP-2
### Chart
| Category | |
|---|---|
| C | 1.0 |
| INPP-1 | 3.369837969400105 |
| INPP-2 | 5.15871672759937 |
INPP4B
Fold Change
COX-2
Tubulin
Additional Figure 4. INPP4B expression inhibits expression of COX-2.
(A and B) PC-3 cells from Tet-On clone #14 and the negative for INPP4B clone were cultured for 2 days ± 0.5 μg/ml doxycycline in full medium. RNA was extracted and analyzed for expression of INPP4B (A) and COX-2 (B) by quantitative PCR and normalized to 18S. Data are presented as means ± SEM. * P<0.05, ***P<0.0001, two-tailed Student’s t test. (C) PC-3 control and inducible clones were cultured for 2 days ± 0.5 µg/ml doxycycline in serum-containing media. Proteins were extracted and the expression of FLAG-INPP4B, COX-2, and tubulin was analyzed by Western blotting. Bar graph, Expression of COX-2 (fold-change) was quantified by densitometry relative to tubulin and normalized to no doxycycline for each clone (expressed as 1.0). (D) LNCaP cells were transfected with either noncoding control (Ctrl) or 2 independent INPP4B-specific siRNAs (INPP-1 or INPP-2). Cells were grown for 48 hours in complete medium and cellular protein extracts were analyzed by Western blotting for INPP4B, COX-2 and tubulin. Bar graph, COX-2 protein levels were quantified by densitometry, normalized to tubulin, and fold change in expression levels was determined relative to control siRNA transfected cells (1.0). Data in panels C and D were obtained three times and representative experiment shown.
