# Supplementary material for: INPP4B suppresses prostate cancer cell invasion
Source: Cell Commun Signal. 2014 Sep 25;12:61. doi: 10.1186/s12964-014-0061-y (PMC4181726; doi:10.1186/s12964-014-0061-y)
Supplement: Additional file 5: — BIRC5 expression increases after castration in LTL-418 xenograft line. A. BIRC5 IHC staining of LTL-418 xenograft tissue harvested before castration. The staining is nuclear ranging form medium to high intensity. B. BIRC5 staining of LTL-418 xenograft tissue harvested 1 week after castration. C. BIRC levels in LTL-418 xenograft tissue 3 weeks after castration showing high level of staining in absolute majority of nuclei. [file 12964_2014_61_MOESM5_ESM.pptx]

## Slide 1
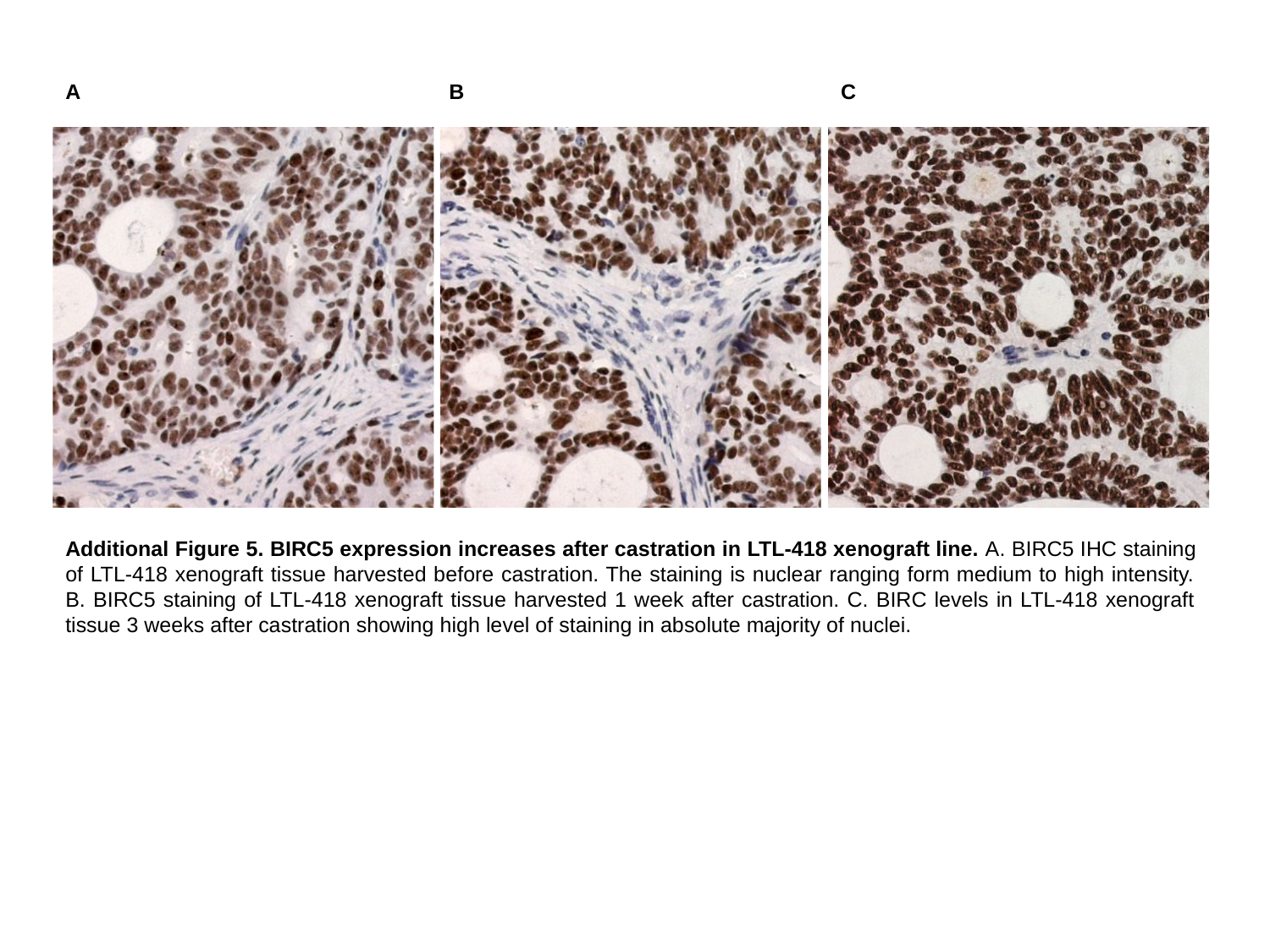

A
B
C
Additional Figure 5. BIRC5 expression increases after castration in LTL-418 xenograft line. A. BIRC5 IHC staining of LTL-418 xenograft tissue harvested before castration. The staining is nuclear ranging form medium to high intensity. B. BIRC5 staining of LTL-418 xenograft tissue harvested 1 week after castration. C. BIRC levels in LTL-418 xenograft tissue 3 weeks after castration showing high level of staining in absolute majority of nuclei.
